# Myocardial Pathology in COVID-19-Associated Cardiac Injury: A Systematic Review

**DOI:** 10.3390/diagnostics11091647

**Published:** 2021-09-08

**Authors:** Aniello Maiese, Paola Frati, Fabio Del Duca, Paola Santoro, Alice Chiara Manetti, Raffaele La Russa, Marco Di Paolo, Emanuela Turillazzi, Vittorio Fineschi

**Affiliations:** 1 Department of Surgical Pathology, Medical, Molecular and Critical Area, Institute of Legal Medicine, University of Pisa, 56126 Pisa, Italy; aniello.maiese@unipi.it (A.M.); alicechiara812@gmail.com (A.C.M.); marco.dipaolo@unipi.it (M.D.P.); emanuela.turillazzi@unipi.it (E.T.); 2 IRCSS Neuromed Mediterranean Neurological Institute, 86077 Pozzilli, Italy; paola.frati@uniroma1.it (P.F.); raffaele.larussa@unifg.it (R.L.R.); 3 Department of Anatomical, Histological, Forensic and Orthopaedic Sciences, Sapienza University of Rome, Viale Regina Elena 336, 00161 Rome, Italy; fabio.delduca@uniroma1.it (F.D.D.); paola.santoro@uniroma1.it (P.S.); 4 Section of Legal Medicine, Department of Clinical and Experimental Medicine, University of Foggia, 71122 Foggia, Italy

**Keywords:** COVID-19, SARS-CoV-2, postmortem, autopsy, heart, heart failure, myocarditis

## Abstract

Coronavirus disease 2019 (COVID-19) can potentially affect all organs owing to the ubiquitous diffusion of the angiotensin-converting enzyme II (ACE2) receptor-binding protein. Indeed, the SARS-CoV-2 virus is capable of causing heart disease. This systematic review can offer a new perspective on the potential consequences of COVID-19 through an analysis of the current literature on cardiac involvement. This systematic review, conducted from March 2020 to July 2021, searched the current literature for postmortem findings in patients who were positive for SARS-CoV-2 by combining and meshing the terms “COVID-19”, “postmortem”, “autopsy”, and “heart” in titles, abstracts, and keywords. The PubMed database was searched following the Preferred Reporting Items for Systematic Reviews and Meta-Analyses (PRISMA) guidelines. Sixteen papers met the inclusion criteria (case reports and series, original research, only English-written). A total of 209 patients were found (mean age (interquartile range (IQR)), 60.17 years (IQR, 54.75–70.75 years); 122 men (58.37%, ratio of men to women of 1:0.7%)). Each patient tested positive for SARS-CoV-2. Death was mainly the result of respiratory failure. The second most common cause of death was acute heart failure. Few patients specifically died of myocarditis. Variables such as pathological findings, immunohistochemical data, and previous clinical assessments were analyzed. Main cardiac pathological findings were cardiac dilatation, necrosis, lymphocytic infiltration of the myocardium, and small coronary vessel microthrombosis. Immunohistochemical analyses revealed an inflammatory state dominated by the constant presence of CD3+ and CD8+ cytotoxic lymphocytes and CD68+ macrophages. COVID-19 leads to a systemic inflammatory response and a constant prothrombotic state. The results of our systematic review suggest that SARS-CoV-2 was able to cause irreversible changes in several organs, including the heart; this is reflected by the increased cardiac risk in patients who survive COVID-19. Postmortem analysis (including autopsy, histologic, and immunohistochemical examination) is an indispensable tool to better understand pathological changes caused by emerging diseases such as COVID-19. Our results may provide more information on the involvement of the heart in COVID-19 patients.

## 1. Introduction

Coronaviruses have always coexisted with humans; however, in 2020, a new severe acute respiratory syndrome coronavirus (SARS-CoV) developed and caused a worldwide pandemic, as the World Health Organization (WHO) declared in March 2020 [1]. SARS-CoV-2 caused a new infectious disease called coronavirus disease 2019 (COVID-19). SARS-CoV-2 is an ssRNA virus belonging to the family of Coronaviridae. The viral genome consists of 27 open reading frames (ORFs) and codes for 27 proteins [2], 4 of which are major proteins and 8 accessory proteins [3]. The most important one, the spike protein, is functionally divided into the S1 and S2 domain; the first domain is crucial for the binding of angiotensin-converting enzyme II (ACE2) on the endothelial cells of the lung. The S2 domain is important for cell membrane fusion [4]. The main feature of SARS-CoV-2 is its high airborne transmission capability [5].

The natural course of severe COVID-19 consists of a primordial phase of airborne transmission, a variable period of viral replication or flulike symptoms, and then an inflammatory dominant phase [6]. The last phase leads to an aberrant host immune response mediated by natural killer (NK) cells, macrophages, and T lymphocytes. This systemic hyperinflammatory response involves the endothelial cells of the lungs and acute respiratory distress syndrome (ARDS), eventually leading to death from respiratory failure [7]. Considering that ACE2 is diffusely expressed among various organs [8], it is commonly accepted that SARS-CoV-2 can and does affect different organs. Therefore, COVID-19 is considered a systemic disease that involves the lungs, heart, kidneys, and liver. During clinical evaluations of patients with COVID-19, it is usual to find evidence of acute respiratory disease syndrome (71%), cardiac injury (33%), acute kidney injury (20%), and liver dysfunction (15%) [9].

Heart involvement correlates to a high mortality rate [10,11]. Specifically, high blood troponin levels strictly relate to higher mortality rates, as confirmed by prospective observational cohort studies [12].

Autopsy data and histopathological analysis of cardiac involvement may help to understand COVID-19 pathophysiology and its clinical features [13]. The aim of the present study was to evaluate the current knowledge about postmortem cardiac findings in patients who died of COVID-19 and to provide a systematic review of existing literature.

## 2. Materials and Methods

This systematic review was conducted from March 2020 to July 2021 and followed the Preferred Reporting Items for Systematic Reviews and Meta-Analyses (PRISMA) guidelines [14]. A systematic literature search of the PubMed database and a critical review of the collected studies were conducted. The database was searched by combining and meshing the terms “COVID-19”, “postmortem”, “autopsy”, and “heart” in titles, abstracts, and keywords. The references of all identified articles were examined and cross-referenced to further isolate the relevant literature. A methodologic appraisal of each study was conducted including an evaluation of bias. The data collection process included study selection and data extraction. This study was exempt from an institutional review board approval as it did not involve human subjects. A waiver of patient consent was provided due to the use of deidentified patient data.

**Figure 1:**
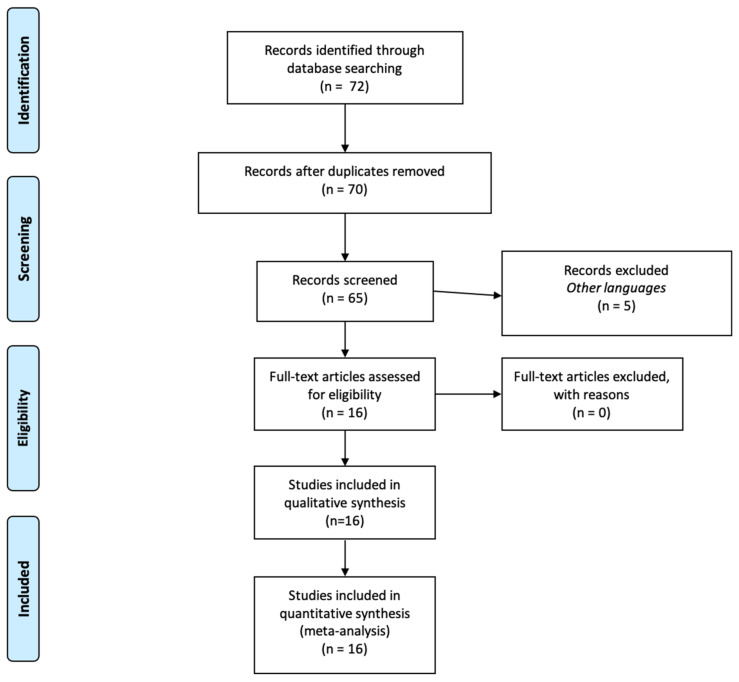
Research results from 70 screened articles. Inclusion criteria included case reports, research articles, autopsy series reports, and papers written in the English language.

Inclusion criteria included case reports, research articles, and autopsy series reports, and papers written in the English language. In Figure 1, a flowchart of our review method is shown.

Regarding immunohistochemistry considerations, the presence or absence of immune cells in the myocardium and myocardial vessels was highlighted. Myocardial SARS-CoV-2 presence was considered positive when viral RNA was detected in the myocardium through RNAscope^®^ (Advanced Cell Diagnostics Inc. Leica, Nussloch, Germany) or alternative RNA in situ hybridization (RNA-ISH) methods, immunohistochemical stains, or when electron microscopy provided evidence of viral particles.

## 3. Results

Literature containing information on the role of SARS-CoV-2 in heart disease was limited but well represented. We detected a total of 72 articles, as reported in the PRISMA flowchart. After reading the abstracts, we screened the remaining articles through a critical revision of the entire datasheet (when available).

After the screening was complete, we selected 16 articles using additional criteria, such as excluding reviews to avoid including the same article twice; or when the article did not provide clinical and laboratory details that we considered essential for consistency with other papers. We decided to include only case reports, research articles, and autopsy series reports.

A total of 209 patients (mean age (interquartile range (IQR)), 60.17 years (IQR, 54.75–70.75 years); 122 men (58.37%)) were identified, with a ratio of men to women of 1:0.7. Each patient tested positive for SARS-CoV-2. Almost all patients had previous comorbidities including chronic obstructive pulmonary disease (COPD), diabetes, hypertension, chronic kidney disease (CKD), hypertrophic cardiomyopathy, ischemic heart disease, atrial fibrillation, cancer, and dementia. A summary of the found articles is reported in Table 1, while the Section 3.1 “Autopsy data” a brief description of the pathological findings described in the papers is provided.

### 3.1. Autopsy Data

In November 2020, Lindler et al., published a cohort study of 39 patients who died of COVID-19–related ARDS [15]. No one died of fulminant myocarditis. The authors evaluated cardiac involvement at autopsy and found that only 15 hearts tested positive for SARS-CoV-2 using RNAscope. Moreover, through immunochemical analysis, they discovered the expression of multiple proinflammatory genes which led to a cytokine storm and the expression of CD3, CD45RO, and CD68. There was diffuse SARS-CoV-2 involvement (>1000 copies) in five hearts associated with a myocardial inflammatory storm. Despite data scarcity, it seems that SARS-CoV-2 infection correlates with a massive inflammatory response.

Fox et al., performed a cohort study of 22 COVID-19-related deaths and published their findings in September 2020 [16]. Macroscopic analysis in 9 of 22 hearts showed a severe right ventricular dilatation due to pulmonary hypertension caused by COVID-19 pneumonia. Furthermore, transmission electron microscopy (TEM) examination of six hearts revealed a large and diffuse viral invasion, in association with CD8+ lymphocytes near the endothelial cells of small vessels.

In September 2020, Jacobs et al., described the case of a man aged 48 years who died of fulminant myocarditis [17]. The aim of the study was to demonstrate the role of ferroptosis (programmed cell death dependent on iron and the accumulation of lipid peroxide) induced by COVID-19. The patient had a progressive multiorgan failure (MOF). In particular, the authors stated that the patient had kidney failure secondary to heart failure. This patient case fulfilled the Dallas criteria for a myocarditis diagnosis, demonstrated by a diffuse muscular and vascular CD3+ lymphocytic invasion [18].

Then, in January 2021, Pellegrini et al., reported a series of 40 autopsies in which the subjects died primarily of respiratory failure (90%) and secondarily of vascular ischemic disease (10%) [19]. The authors sampled the myocardium for histologic processing and analyzed the coronary artery aspirates. Microscopy revealed focal necrosis in 35% of cases, with a significant relationship (*p* < 0.001) between myocardial necrosis and concomitant microthrombi.

In January 2020, Gauchotte et al., described the postmortem heart findings of a 69-year-old man who died of heart failure without lung involvement [20]. Histology findings demonstrated inflammatory infiltration of the cardiac muscle along with positivity for SARS-CoV-2 using RNAscope. Noteworthy, RNAscope analysis of the lung tissue was negative for COVID-19.

Further investigation of infectious myocardial changes was performed by Bulfamante et al., in December 2020 [21]. The authors studied myocardial sections, which had tested positive for the presence of SARS-CoV-2, to investigate changes in the ultrastructure of the myocardium. The main features found were cardiac edema, loss of regular morphology, and cardiomyocyte swelling. Interestingly, SARS-CoV-2 viral particles and sarcomere ruptures were co-localized.

Del Nonno et al., published a report in November 2020 that investigated patients who died of COVID-19 but had negative heart samples [22]. They examined a small cohort of nine hospitalized patients who died of cardiogenic shock [6] or sudden death [13]. They were all receiving pharmacologic treatment for SARS-CoV-2-related pneumonia. The aim of this study was to evaluate direct and indirect involvement of COVID-19-related cardiac damage through a series of autopsies. The lymphomononuclear infiltrates CD68- and CD45Ro+ were found, surrounded by focal necrosis of adjacent myocytes; these were indicative of myocarditis. Six patients who died of acute heart failure had necrosis of the vessel walls. Inflammatory infiltrates were found in the subepicardial ganglia of three patients who died of sudden cardiac death. Molecular tests ruled out the presence of viral infections—including SARS-CoV-2—as the cause of myocarditis. The authors concluded that the inflammatory component was a consequence of the initial damage from the viral infection; the virus may have triggered a secondary and independent phase of inflammation.

In September 2020, Basso et al., examined a total of 22 hearts, 3 of which were affected by myocarditis and 19 by lymphocytic infiltration [23]. This study revealed an abundant number of CD68+ macrophages in the hearts of patients who died of COVID-19. As a matter of fact, CD68+ macrophages and CD3+ lymphocytes were more abundant in the myocarditis group than in the control group. The presence of CD68+ cells may suggest a systemic elevation of proinflammatory cytokines, such as interleukin-6 (IL-6) and tumor necrosis factor-α (TNF-α), during COVID-19 infection; this could explain the recruitment of macrophages.

Hanley et al., reported an autopsy series in August 2020 that focused on cardiac findings: five hearts of patients who died of COVID-19 had thrombi in the microcirculation, and one revealed macroscopic evidence of acute right coronary artery thrombosis [24]. Necrosis associated with high viral load was found in two myocardial samples.

Rapkiewicz et al., performed seven autopsies of patients who tested positive for SARS-CoV-2 [25] in June 2020. There was only one patient with evidence of predominant CD4+ lymphocytic infiltration. However, the main finding was the presence of fibrin microthrombi regardless of anticoagulation therapy.

In July 2020, Cîrstea et al., published a case of a woman aged 30 years who died of COVID-19-related pneumonia at Drobeta-Turnu Severin hospital in Romania [26]. The patient had presented with breathing difficulties, high fever, chest pain, and loss of appetite. At autopsy, the forensic pathologist found bilateral pneumonia and right ventricular dilatation, which lead to respiratory failure. Heart analysis showed leukostasis with thrombi formation and massive interstitial edema that obliterated the intercalated discs. There was no evidence of lymphocyte or macrophage infiltration.

In August 2020, Bradley et al., described a cohort of older patients with multiple comorbidities [27]. According to recent literature which asserts the high mortality rate of patients with comorbidities, the authors did not describe specific pathological findings [28,29]. They only reported nonspecific findings, such as fibrosis and hypertrophy. In eight patients, the authors found lymphocytes surrounding necrotic myocytes. No immunochemistry analysis was performed.

In May 2020, Duarte-Neto et al., presented the histopathological features of 10 minimally invasive autopsies (MIA-US) [30,31]. Histologic observation of the cardiac tissue revealed cardiomyocyte hypertrophy, edema, and myocardial fibrosis in nine patients and previous myocardial infarction in four patients. Further, the authors found microthrombi in the arterioles of two patients and evidence of myocarditis. They concluded that MIA-US was a safe, rapid, and effective procedure for obtaining tissue samples for the study of severe COVID-19.

**Table 1:** Summary of data obtained from the 16 found articles.

Authors	Number of Cases	Age	M:F	Cardiac Death	Cardiac Infection	Macroscopic			Microscopic			IHC
						Weight	Ventricular Dilatation/ Myocyte Hypertrophy	Necrosis		Lymphocytic Infiltration Parenchyma	Microthrombi/ Cardiac Vessels Involvement	
Lindner et al. 2020 [15]	39	85 (78–89)	16:23	1 Prior HF, 38 ARDS	15 (+) interstitial cells within the cardiac tissue	Not reported	None	None		0	-	Yes
Fox et al. 2020 [16]	22	68.5 (44–79)	10:12	0	16	340 to 1010 g	9 RVD Se R:L >1:1 Right Hypertrophy	Individual cell dropout/necrosis/apoptosis		0	-	Yes
Jacobs et al., 2020 [17]	1	48	1	1 Fulminant Myocarditis due to COVID-19	1	605	1	piecemeal necrosis		1	-	Yes
Pellegrini et al. 2020 [19]	40	74	29:11	3 HF, 37 RF	8	Not reported	Not reported	14 (3 infarction, 11 focal necrosis)		9	9	No
Gauchotte et al. 2021 [20]	1	69	1	1 Fulminant myocarditis due to COVID-19	1	403	none	None		1	-	Yes
Bulfamante et al., 2020 [21]	6	59.5 (54–69)	1:5	6 RF	6	410–750	1 LVH (prior), 6 RVD (lung overload)	6		6	-	Yes
Del Nonno et al., 2020 [22]	9	70 (35–92)	7:2	9 (6 shock, 3 sudden death)	0	450	No supplementary available	9		9	9	No
Basso et al. 2020 [23]	21	69 (44–86)	15:6	1 HF, 1 Sudden death, 19 RF	Not reported	Not reported	Not reported	4		3 criteria /11 infiltration	3	Yes
Hanley et al. 2020 [24]	10	73 (52–79)	6:3	9 RF	2	450 (312–513)	4 LVH	1		0	6	No
Rapkiewicz et al., 2020 [25]	7	57 (44–65)	3:4	7 RF	Not reported	Not Reported	Not reported	3		1	7	No
Cîrstea et al., 2020 [26]	1	30	0:1	1 HF	Not reported	Not reported	Not reported	0		1	1	No
Bradley et al., 2020 [27]	14	73.5 (42–84)	6:8	1 VF, 1 LVF, prior 12 RF	0	Not reported	13 CH	1		1	0	No
Duarte-Neto et al., 2020 [30]	10	69 (33–83)	5:5	10 RF	Not reported	Not reported	9 CH	0		2	2	No
Schaller et al., 2020 [32]	10	69 (64–90)	7:4	Not reported	Not reported	Not reported	Not reported	Not reported		4	0	No
Buja et al., 2020 [33]	3	48 (34–62)	3:0	2RF, 1 SD	Not reported	720 (420–1070)	2CH	0		2	0	No
Bois et al., 2021 [34]	15	78 (71–86)	12:3	Not reported	5	443.1 (286.3–545.0)	Not reported	9		5	12	Yes

HF—Heart Failure; RF—Respiratory failure; CH—Cardiac Hypertrophy; LVH—Left Ventricular Hypertrophy; RVD—Right Ventricular Disfunction; IHC—immunohistochemistry.

Schaller et al., wrote a letter in June 2020 regarding the features of 10 COVID-19 autopsies [32]. Cardiac involvement was minimal, as they reported only mild lymphocytic myocarditis in four cases. The main characteristics included diffuse alveolar damage (DAD), which was found in both ventilated and nonventilated patients; this suggests the idea that DAD is not only a consequence of noninvasive ventilation.

In April 2020, Buja et al., described three autopsies conducted at Houston hospital [33]. They reported the findings of patients who were treated earlier during the onset of the pandemic. Immunochemistry was performed in each case. The authors found a large lymphocytic infiltration in the myocardium and pericardium. Specifically, tissue staining revealed CD8+–predominant inflammation, with diffuse CD68+ macrophages. The cardiac conduction system was not involved.

Bois et al., recently described histopathological, immunohistochemical, ultrastructural, and molecular findings of the heart in patients who died of active or cured SARS-CoV-2 infection [34]. One-third of the patients with COVID-19 showed myocarditis but this, due to its limited extent, could not account for the clinical picture. The finding of cardiac fibrin microthrombi in the absence of acute ischaemic lesions was the most significant finding of this study. No viral particles were identified within cardiac myocytes by transmission electron microscopy.

**Figure 2:**
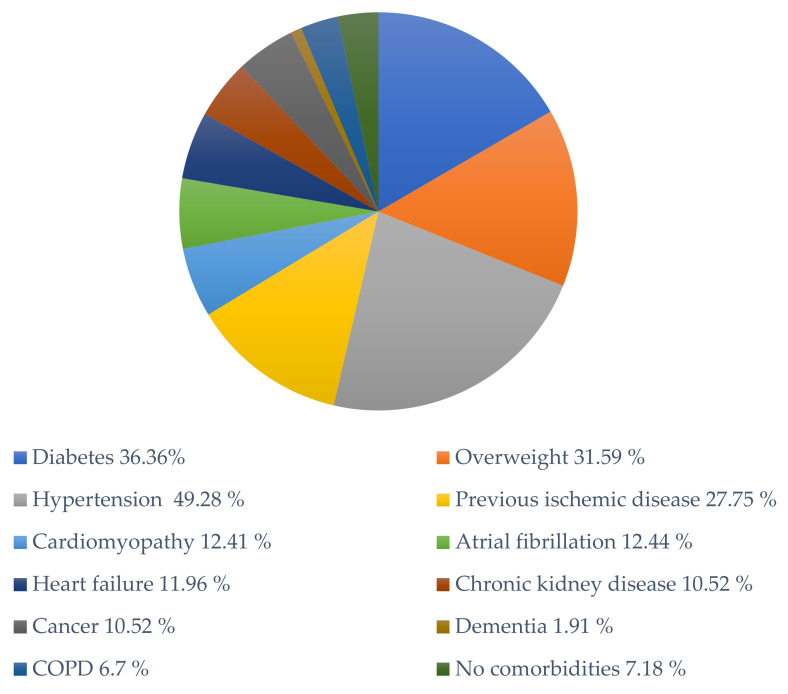
Comorbidities’ frequency in the subjects described in the articles included in this review (total *n* = 209). COPD indicates chronic obstructive pulmonary disease.

Immunohistochemistry results and the comorbidities of the patients are summarized in Table 2 and Table 3, respectively. Figure 2 shows the percentage of comorbidities observed among all the subjects (209).

## 4. Discussion

Post-mortem studies on COVID-19 deaths revealed various organs alterations. The lungs were extensively described. They appeared edematous and their weight was generally increased. Microscopically, the main characteristic was exudative and proliferative DAD, less frequently with fibrotic features [13]. The other organs manifested unspecific findings, often related to lymphocyte infiltration, small vessels inflammation, and microthrombi [13]. Concerning heart involvement, it may be interesting to take into consideration the alterations caused by other human coronaviruses. Myocardial damage in SARS-CoV infection consists of edema in both the myocardial stroma and vascular walls, in addition to atrophy of the cardiac muscle fibers [35]. In Middle East respiratory syndrome (MERS), the other well-known betacoronavirus disease, cardiac involvement is secondary to lung inflammation that occurs in bronchiolitis obliterans organizing pneumonia [36]. In contrast, in this study, we found substantial cardiac involvement in SARS-CoV-2 infections.

In this systematic review, we evaluated the cardiac features of 209 hearts, the majority of which were not analyzed with immunostaining.

**Figure 3:**
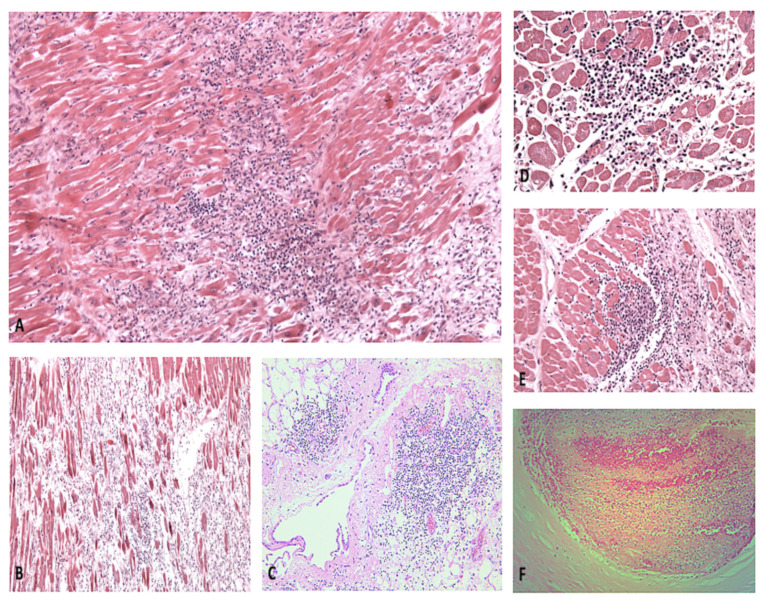
(**A**,**B**) Myocarditis with extensive myocyte necrosis, at different developmental stages (H&E, 60× and 20×, respectively). (**C**) Massive lymphocytic infiltration of the pericardium and perivascular area (H&E, 40×). Foci of acute myocyte necrosis (**D**) and regions undergoing tissue repair (*E*) (H&E, 100× and 80×, respectively). (**F**) Acute coronary thrombosis (H&E, 200×).

According to our results, most patients died of respiratory failure, and less than 10% died of cardiac failure. One of the main features was right heart dilatation due to pulmonary overload. Recurrent microscopic findings included necrosis, lymphocytic infiltration of the myocardium, and microthrombosis of the small cardiac vessels (Figure 3), as it was described in other organs [13].

**Figure 4:**
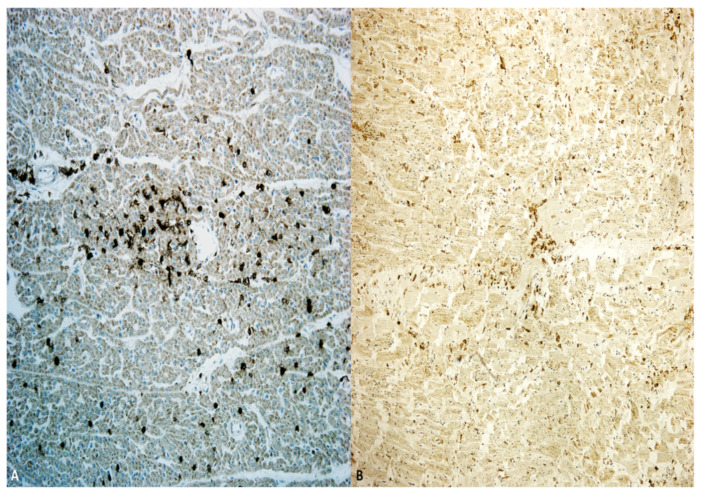
Myocarditis: immunohistochemistry demonstrating CD68 macrophage population (**A**) and CD8 expression (**B**).

When histologic infiltrates were studied, they revealed a large CD3+ and CD8+ cytotoxic lymphocytic infiltration and the presence of CD68+ macrophages (Figure 4).

Cellular immunity has been demonstrated to play a fundamental role in COVID-19 host response, so the relative abundance of CD3 + cells in the tissues is not surprising [37]. In our results, the ratio of CD4 to CD8 was generally less than 1, as it happens in other viral infections [38]. On the contrary, other studies performed on COVID-19 patients showed that the CD4:CD8 ratio was not reduced, whereas a reduction of the total number of both CD4+ and CD8+ lymphocytes in the blood was observed [7,39]. It may depend on the different localization of CD8+ T cells, which may be mainly located within the tissues showing a higher positivity at immunohistochemical staining. It must be taken into account also that in some studies the CD4:CD8 ratio was increased and correlated with the severity of the disease [40,41]. Various hypotheses may be advanced to explain these differences among the studies. At first, the CD4:CD8 ratio may depend on the different stages of the disease, with a progressive decrease in CD8+ T cells. For sure, more studies are needed to deepen the immunological pattern in COVID-19 patients and our review does not include enough cases to allow further speculations.

Concerning the pathophysiology of the cardiac involvement, we hypothesize both direct organ damage mediated by SARS-CoV-2 and an indirect insult due to the subsequent inflammatory response. The inflammatory cascade leads to multiple proinflammatory agents, such as cytokines and CD8 cytotoxic cells, that induce tissue damage [42]. Halushka and Vander Heide described a low incidence of histopathologic myocarditis interpreting cardiac symptoms in COVID-19 patients as consequences of other stressors such as endothelial cell activation, cytokine storms, or electrolyte imbalances [43]. According to the current model of SARS-CoV-2 tropism for the ACE2 receptor, the primary cause of COVID-19–related death is respiratory failure due to atypical severe ARDS [7,44]. ACE2 is expressed also in the heart, and it also may be the target of SARS-CoV-2 in this organ, inducing cardiac injury [45,46,47]. Regarding the cardiac alterations in clinical studies, many Authors described troponin levels elevation and echocardiographic abnormalities [45,48]. It is not clear if these findings should be interpreted as direct myocardial damage since, as our results also show, usually COVID-19 patients have cardiovascular comorbidities that may complicate the clinical picture [49]. Lassen et al., performed a prospective multicentre cohort study, collecting heart ultrasound data from 214 hospitalized COVID-19 patients. They found that the left and right ventricular systolic function was significantly decreased, even correcting their results for previous cardiac comorbidities [50]. However, echocardiography remains a crucial investigation in monitoring COVID-19 patients and detecting cardiac involvement [51].

Postmortem findings, in particular histological and immunohistochemical analysis, are fundamental to better understand the pathophysiological mechanism of cardiac involvement in COVID-19, and consequently to develop future disease management strategies [52,53,54].

## 5. Conclusions

Currently, there is minimal standardized and validated literature available about the cardiac implications of COVID-19. Our study results suggest a high rate of cardiac involvement in such cases. In the future, COVID-19 outcomes should be further studied to better individualize therapeutic strategies. Postmortem examination and histologic and immunohistochemical analysis of patients who died of COVID-19 are pivotal to the development of future methods of disease management and to provide useful tools with the goal of controlling viral spread in a pandemic.

As our results demonstrated, cardiac inflammation, mostly represented by lymphocytic infiltration and perivascular aggregates, is not an unusual finding in COVID-19 deaths. Besides, further studies are needed to disclose the possible therapeutic applications of these findings. The prevention or attenuation of the cytokine storm and endothelial cell activation may be helpful to mitigate cardiac involvement [55]. Indeed, a better understanding of cardiac involvement pathophysiology and the pathological pattern is needed and autoptic studies on COVID-19 deaths should be further promoted [13,56].

Further studies, specifically performed on forensic casuistry, are needed for the thorough evaluation of the etiology of death of patients infected with COVID-19 and for the identification of useful pathognomonic signs of cardiac involvement.

## Figures and Tables

**Table 2:** Immunohistochemistry results as reported by authors.

Authors	Number of Cases	Tissue RNA Scope or TEM	CD3	CD4	CD8	CD4/CD8	CD68
Lindner et al., 2020 [15]	39	24	21	-	-	1	19
Fox et al., 2020 [16]	22	6	6	0	6	<1	-
Jacobs et al., 2020 [17]	1	1	1	1	1	1:7	1
Pellegrini et al., 2020 [19]	40	40	8	-	-	-	-
Gauchotte et al., 2021 [20]	1	1	1	1	1	1:9	1
Bulfamante et al., 2020 [21]	6	6	6	-	-	-	-
Del Nonno et al., 2020 [22]	9	-	-	-	-	-	-
Basso et al., 2020 [23]	21	21	21	2	19	<1	18
Hanley et al., 2020 [24]	10	9	-	-	-	-	-
Rapkiewicz et al., 2020 [25]	7	7	1	1	1	<1	0
Cîrstea et al., 2020 [26]	1	1	-	-	-	-	-
Bradley et al., 2020 [27]	14	-	-	-	-	-	-
Duarte-Neto et al., 2020 [30]	10	-	-	-	-	-	-
Schaller et al., 2020 [32]	10	10	-	-	-	-	-
Buja et al., 2020 [33]	3	2	2	2	2	2:1	1
Bois et al., 2021 [34]	15	15	-	-	-	-	-

**Table 3:** Anamnesis findings of patient comorbidities. COPD indicates chronic obstructive pulmonary disease.

	Number of Cases	Diabetes	Overweight	Hypertension	Previous Ischemic Disease	Cardiomyopathy	Atrial Fibrillation	Heart Failure	Chronic Kidney Disease	Cancer	Dementia	COPD	No Comorbidities
Lindner et al., 2020 [15]	39	17	-	17	17	-	-	1	-	-	-	0	-
Fox et al., 2020 [16]	22	22	11	9	18	1	9	2	2	4	0	0	0
Jacobs et al., 2020 [17]	1	-	1	1	-	1	-	0	0	0	0	0	0
Pellegrini et al., 2020 [19]	40	11	40	29	8	0	3	0	7	5	0	0	0
Gauchotte et al., 2021 [20]	1	1	0	1	1	1	1	0	1	0	0	0	0
Bulfamante et al., 2020 [21]	6	0	0	2	1	1	0	0	0	0	0	0	3
Del Nonno et al., 2020 [22]	9	-	-	-	-	2	0	6	0	1	1	0	3
Basso et al., 2020 [23]	21	7	-	16	3	7	4	7	2	4	-	1	0
Hanley et al., 2020 [24]	10	0	0	3	0	0	0	0	0	0	0	3	4
Rapkiewicz et al., 2020 [25]	7	5	5	6	1	1	0	0	1	2	0	1	1
Cîrstea et al., 2020 [26]	1	0	0	0	0	0	0	0	0	0	0	0	1
Bradley et al., 2020 [27]	14	5	4	6	4	13	8	8	5	2	2	3	0
Duarte-Neto et al., 2020 [30]	10	5	-	5	4	5	-	-	1	1	-	3	0
Schaller et al., 2020 [32]	10	2	2	7	1	3	-	-	3	3	1	2	0
Buja et al., 2020 [33]	3	1	3	1	0	1	1	1	0	0	0	1	0
Bois et al., 2021 [34]	15	-	-	-	-	-	-	-	-	-	-	-	3
Total	209	76	66	103	58	36	26	25	22	22	4	14	15

## Data Availability

No new data were created or analyzed in this study. Data sharing is not applicable to this article.
